# Comparing Gene Trees and Genome Trees: A Cobweb of Life?

**DOI:** 10.1371/journal.pbio.0030347

**Published:** 2005-08-30

The tree of life has long served as a useful tool for describing the history and relationships of organisms over evolutionary time. One species is represented as a branching point, or node, on the tree, and the branches represent paths of descent from a parental node. The tree diagram carries an implicit assumption that genes are transferred vertically, from parent to child, and that all the genes in a new species come from the ancestral species. In theory, one should be able to trace the origin of each gene in a species back to its ancestor. In practice, however, the ancestral gene is rarely available, so researchers look for the gene in a closely related species. (These similar genes, which diverge slightly after a speciation event, are called orthologs.)[Fig pbio-0030347-g001]

**Figure pbio-0030347-g001:**
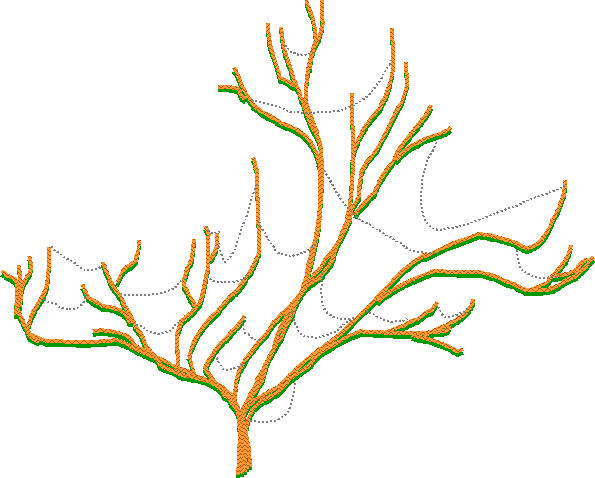
Horizontal gene transfers—gene exchange between non-related organisms—appear commonplace among bacteria, but contribute just small bits of genetic information, leaving the traditional tree of life intact

But as the tools of genome analysis became more refined, searches for similar genes sometimes turned up sequences that belonged to a species on a different branch of the evolutionary tree. Clearly, vertical gene transfer was not the only mechanism of genetic transmission. Organisms, it turns out, can acquire genes from non-ancestral species through a mechanism called horizontal gene transfer (HGT)—think of it as acquiring genes from your neighbor instead of your parents. Such genetic exchanges, most common among bacteria and other microbes, are not represented in the tree of life—no single branch connects the two unrelated species. Initial studies suggested that HGT events were extremely common, prompting some to say it was time to replace the tree with a netlike diagram. Other studies have since suggested that methods used to calculate HGT overestimated its frequency: researchers detect HGT events by finding inconsistencies between gene trees and organism, or whole-genome, trees, but statistical errors can artificially increase the number of HGT events.

In a new study, Fan Ge, Li-San Wang, and Junhyong Kim estimate the frequency of HGT events by using a novel statistical method to compare the gene trees and whole-genome trees of microbes. Their method solves the statistical problem by directly testing for discrepancies between trees that arise from statistical error versus true HGT events. Analyzing over 40 microbial genomes, Kim and colleagues estimate that HGT infiltrates just 2% of the average microbial genome. Even when relatively common, the authors conclude, HGT events do not disrupt the integrity of the tree of life, contributing just small bits of genetic material, “much like cobwebs on tree branches.”

To construct both gene trees and a whole-genome tree for the microbes, the authors selected core sets of orthologous gene groups from the NIH database of clusters of orthologous genes. (Clusters are derived by comparing protein sequences encoded in complete genomes, which represent major lineages on the evolutionary tree. Each cluster corresponds to an ancient, conserved protein domain.) Kim and colleagues created gene trees for each cluster of orthologous genes they selected, then created whole-genome trees from the gene trees and compared each gene tree to the whole-genome tree, using their new method. HGT events were detected when two species appeared close together on a gene tree but far apart on the whole-genome tree. Overall, just over 11% of the orthologous gene clusters showed statistically significant HGT events, with HGTs accounting for about 2% on average of each of the 40 microbial genomes.

Altogether, these results suggest that HGT is not as common as once thought. And even when large-scale HGT events do occur—which Kim simulated in a previous study—they do not obscure the evolutionary path of most genes and lineages. If you imagine a tree with 10,000 taxa, the authors explain, and 1,000 HGTs per genome across all the taxa, the HGTs would form “extremely thin connections like cobwebs,” leaving the backbone of the tree intact. Infrequent though it may be, HGT likely has some impact on the evolutionary history of life—impacts that advances in genome analysis technology may help uncover. —*Liza Gross*

